# Congenital Dyserythropoietic Anemia Type II Caused by a New *SEC23B* Sequence Variant

**DOI:** 10.1155/crh/7158943

**Published:** 2026-09-02

**Authors:** Geir Erland Tjønnfjord, Arne Loraas, Signe Spetalen, Liv Toril Nygård Osnes, Kristian Tveten

**Affiliations:** ^1^ Department of Hematology, Oslo University Hospital, Oslo, Norway, oslo-universitetssykehus.no; ^2^ Institute of Clinical Medicine, University of Oslo, Oslo, Norway, uio.no; ^3^ Department of Internal Medicine, Vestre Viken Hospital Trust, Drammen, Norway, vestreviken.no; ^4^ Department of Pathology, Oslo University Hospital, Oslo, Norway, oslo-universitetssykehus.no; ^5^ Department of Immunology and Transfusion Medicine, Oslo University Hospital, Oslo, Norway, oslo-universitetssykehus.no; ^6^ Department of Medical Genetics, Telemark Hospital Trust, Skien, Norway, sthf.no

**Keywords:** CDA Type II, molecular genetics, *SEC232B*sequence variant

## Abstract

Congenital dyserythropoietic anemias (CDAs) include a heterogeneous group of conditions characterized by insufficient erythropoiesis giving rise to monolinear cytopenia. Based on the morphological features of erythroblasts, three major subtypes have been established: CDA Type I, CDA Type II, and CDA Type III. CDA Type II is the most common type of CDAs, and this disease is characterized by a normocytic anemia with a normal or slightly increased reticulocyte count. The bone marrow is hypercellular with erythroid hyperplasia and binucleated erythroblasts with two nuclei at the same maturation stage. The inheritance is autosomal recessive, and the disease is caused by biallelic mutations in the *SEC28B* gene. More than 100 pathogenetic variants have been identified. Here, we report on three patients from Somalia with CDA Type II caused by a new *SEC23B* sequence variant. They came to attention and were diagnosed in early adulthood. Cases 1 and 2 had mild anemia with signs of hemolysis, and Case 3 had mild anemia with reticulocytosis. The eosin‐5‐maleimide binding test showed reduced binding in all three patients. Molecular genetics disclosed homozygosity for the sequence variant NM_006363.6:c.1580T > C p.(Leu527Ser) in the *SEC23B* gene. A bone marrow aspirate was performed from Case 2, and the smear disclosed morphological abnormalities typical for CDA Type II.

## 1. Introduction

Congenital dyserythropoietic anemias (CDAs) include a heterogeneous group of conditions characterized by insufficient erythropoiesis. Monolinear cytopenia is the rule. Bone marrow morphological abnormalities with erythroid hyperplasia and binuclearity or multinuclearity of late erythroblasts have been the cornerstone of diagnosis. Based on the morphological features of the erythroblasts, CDAs have been classified in three major subtypes: CDA Type I, CDA Type II, and CDA Type III [1]. In more recent years, molecular genetics has become an additional diagnostic tool.

CDA Type II is the most common subtype, and it is characterized by a normocytic anemia with a normal or slightly increased reticulocyte count. The bone marrow is hypercellular with erythroid hyperplasia and binucleated erythroblasts with two nuclei, occasionally three or four, at the same maturation stage [1]. The inheritance is autosomal recessive, and the disease is caused by biallelic mutations in the *SEC23B* gene. More than 100 pathogenetic variants have been identified [1–4]. Here, we report on three patients from Somalia with CDA Type II caused by a new *SEC23B* sequence variant.

## 2. Case Presentation

### 2.1. Cases 1 and 2

**FIGURE 1:**
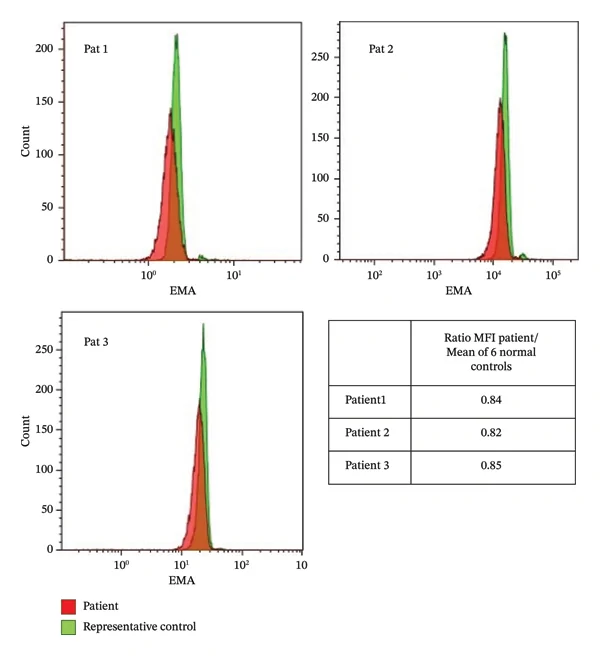
Staining of erythrocytes with eosin‐5′‐maleimide (EMA) in representative healthy blood donor and Patients 1, 2, and 3. Results are given as ratio MFI (mean fluorescence intensity) of patient/mean MFI of 6 normal controls. Cutoff for positivity: < 0.85 positive, 0.85–0.90 borderline, and > 0.9 normal.

At 28 years of age, during her third pregnancy, the proband was referred to a hematological examination due to anemia. Her hemoglobin was 8.7 g/dL (11.7–15.3), erythrocytes 2,9 × 10^12^/L (3.9–5.2), MCH 29 pg (27–33), and MCV 89 fL (82–98). Her other blood cell counts were normal except for reticulocytosis, 180 × 10^9^/L (30–100). There were signs of hemolysis; haptoglobin < 0.1 g/L (0.4–2.1), LD 209 U/L (< 205), and bilirubin 37 μmol/L (5–25). The direct antiglobulin test was negative, and the eosin‐5‐maleimide (EMA) binding test (ratio) was 0.87 and 0.84 (> 0.90) on two occasions one week apart (Figure 1). A blood smear showed anisocytosis and occasional spherocytes and erythroblasts. Splenomegaly was disclosed by an ultrasound examination (longest diameter 16 cm). A diagnosis of hereditary spherocytosis (HS) was made.

Forty‐three months later, she was admitted to hospital with severe anemia (Hb 4.7 g/dL) and reticulocytopenia (32 × 10^9^/L) following 3 days of fever. A primary parvovirus B19 infection was confirmed by positive serology and parvovirus DNA detected in blood. Following this admission, she was referred for reassessment of her hemolytic anemia. The EMA binding test (ratio) was 0,91 at this time, which may be explained by the transfusion of two units of packed red cells two weeks previously. The blood smear disclosed anisocytosis, poikilocytosis, and occasional spherocytes and erythroblasts. Her physiognomy was normal. Molecular genetics disclosed that she was homozygous for the sequence variant NM_006363.6:c.1580T > C p.(Leu527Ser) in the *SEC23B* gene.

**FIGURE 2:**
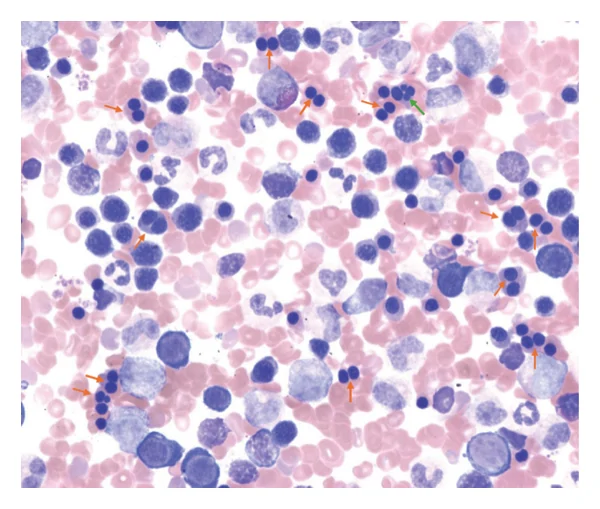
Bone marrow smear from Case 2 prepared by May–Grünwald–Giemsa staining (magnification 630*x*) shows a hypercellular bone marrow dominated by erythropoiesis. There are several binucleated near‐orthochromic erythroblasts (red arrows) and one erythroblast with four nuclei (green arrow).

During this reevaluation, she reported that a 4‐year‐old younger brother (Case 2) also suffered from anemia. He accepted an offer of reassessment of his anemia. His medical history indicated episodes during which the anemia was more pronounced. The laboratory workup is shown in Table 1. A blood smear showed anisocytosis and occasional erythroblasts. Leukocytes and thrombocytes appeared normal, and a bone marrow smear revealed a hypercellular bone marrow with erythroid hyperplasia and several binuclear erythroblasts; approximately 20% of the erythroblasts (Figure 2). Molecular genetics disclosed that he also was homozygous for the same sequence variant in the *SEC23B* gene previously disclosed in his sister. His physiognomy was normal. Their parents are cousins and immigrants from Somalia.

**TABLE 1:** Laboratory characteristics of the patients during steady state.

	Case 1	Case 2	Case 3	Ref. values Females	Ref. values Males
Hemoglobin g/dL	10.7	10.4	11.3	11.7	13.4–17.0
Erythrocytes x10^12^/L	3.6	3.7	3.7	3.9–5.2	4.3–5.7
MCH pg	29.5	29	32	27–33	27–33
MCV fL	100	85	90	82–98	82–98
Reticulocytes x10^9^/L	218	118	128	30–100	30–100
Haptoglobin g/L	< 0.10	0.1	0.8	0.4–2.1	0.4–2.1
LD U/L	386	183	148	< 205	< 205
Bilirubin μmol/L	33	24	75	5–25	5–25
EMA binding test (ratio)	0.84	0.82	0.85	> 0.90	> 0.90
Ferritin μg/L	357	263	188	10–167	29–383

### 2.2. Case 3

At the age of 29 years, she was hospitalized due to abdominal pain. She was icteric, and cholecystolithiasis was suspected. However, this was not confirmed by imaging. Her physiognomy was normal.

She was slightly anemic (hemoglobin 11.3 g/dL) with hyperbilirubinemia (bilirubin 75 μmol/L and conjugated bilirubin 5 μmol/L). Her other laboratory data are shown in Table 1. The direct antiglobulin test was negative, and the EMA binding test was 0.85. A blood smear showed anisocytosis and occasional spherocytes, but no erythroblasts.

Because of increased unconjugated bilirubin, she was tested for Gilbert`s syndrome. She was homozygous (TA7/TA7) in the promotor region of UGT1A1, compatible with Gilbert`s syndrome.

Molecular genetics disclosed that she was homozygous for the sequence variant NM_006363.6:c.1580T > C p.(Leu527Ser) in the *SEC23B* gene.

At the age of 30 years, she had an uncomplicated laparoscopic cholecystectomy. Cholecystolithiasis was confirmed.

She is an immigrant from Somalia.

The EMA binding test was performed as follows. Washed packed erythrocytes (5 μL) from the patient and six healthy controls were incubated with 25 μL of EMA solution (0.5 mg/mL PBS) for 1 hour in the dark and mixed 3–4 times during incubation. After washing with PBS/BSA to remove unbound EMA, the EMA‐labeled erythrocytes were resuspended in PBS/BSA for flow cytometric analysis. The results were the actual median fluorescence intensity (MFI) reading for the patient sample as a ratio by normalization of the test MFI result to the mean MFI value of the normal controls [5].

Whole‐exome sequencing was performed using Twist Exome 2.0 (Twist Bioscience, San Francisco, CA, USA) and NextSeq500 (Illumina, San Diego, CA, USA). We applied a standard bioinformatic approach for base calling (RTA, Illumina), read alignment (BWA, Illumina), and variant detection (best practices variant calling using GATK).

## 3. Discussion

These three individuals with Somalian ancestry presented with moderate normochromic and normocytic anemia with reticulocytosis. They had all experienced episodes of worsening anemia during intercurrent disease. In Case 1, an initial diagnosis of HS was made following a positive EMA binding test. Misdiagnosis between CDA Type II and HS is well known, and CDA Type II is recognized as a disease which characteristically shows reduced EMA binding [1, 6, 7]. Molecular genetics, which was performed to confirm HS, disclosed, however, that she was homozygous for the sequence variant c.1580T > C p.(Leu527Ser) in the *SEC23B* gene. Accordingly, we had to reconsider her initial diagnosis, and in doing so, we examined her brother (Case 2) reported to suffer from anemia. He was also found to be homozygous for the same sequence variant in the *SEC23B* gene. A bone marrow examination revealed erythroid hyperplasia and binucleated erythroblasts typical of CDA Type II (Figure 2). In parallel, another Somalian female, unrelated to the siblings, was evaluated for anemia (Table 1), and she was also found to be homozygous for the same sequence variant in the *SEC23B* gene.

Leu527Ser are in or near the core structures of *SEC23B*, and it likely destabilizes the protein or perturbs folding [2]. The variant is absent from the Genome Aggregation Database, and it is detected in three affected individuals. Bone marrow examination revealed typical CDA Type II findings (ACMG classification: likely pathogenic PP3, PM2, PM3‐P, and PP4‐M). Our studies indicate that CDA Type II in these three individuals is caused by a new *SEC23B* sequence variant. This is the first report on CDA Type II in persons from the Horn of Africa, and the sequence variant disclosed in these three patients may represent a founder mutation.

## Author Contributions

Geir Erland Tjønnfjord and Arne Loraas cared for the patients and collected the clinical data. Liv Toril Nygård Osnes was responsible for flow cytometric studies and created Figure 1. Signe Spetalen was responsible for bone marrow morphology and created Figure 2. Kristian Tveten was responsible for molecular genetics. Geir Erland Tjønnfjord drafted the manuscript, and all coauthors took part in editing the manuscript. Geir Erland Tjønnfjord had full access to all data in the study and takes complete responsibility for the integrity of the data and the accuracy of the data analysis.

## Funding

There was no specific funding for this project.

## Disclosure

All authors have read and approved the final version of the manuscript.

## Consent

Informed consent was obtained from all three patients.

## Conflicts of Interest

The authors declare no conflicts of interest.

## Data Availability

Data and materials will be made available upon reasonable request.
